# Impaired Clearance From the Brain Increases the Brain Exposure to Metoclopramide in Elderly Subjects

**DOI:** 10.1002/cpt.2052

**Published:** 2020-10-14

**Authors:** Martin Bauer, Karsten Bamminger, Verena Pichler, Maria Weber, Simon Binder, Alexandra Maier‐Salamon, Ammar Tahir, Walter Jäger, Helmuth Haslacher, Nicolas Tournier, Marcus Hacker, Markus Zeitlinger, Oliver Langer

**Affiliations:** ^1^ Department of Clinical Pharmacology Medical University of Vienna Vienna Austria; ^2^ Department of Biomedical Imaging and Image‐Guided Therapy Division of Nuclear Medicine Medical University of Vienna Vienna Austria; ^3^ Department of Clinical Pharmacy and Diagnostics University of Vienna Vienna Austria; ^4^ Department of Pharmacognosy University of Vienna Vienna Austria; ^5^ Department of Laboratory Medicine Medical University of Vienna Vienna Austria; ^6^ Laboratoire d'Imagerie Biomédicale Multimodale (BioMaps) CEA CNRS Inserm Service Hospitalier Frédéric Joliot Université Paris‐Saclay Orsay France; ^7^ Preclinical Molecular Imaging AIT Austrian Institute of Technology GmbH Seibersdorf Austria

## Abstract

The antiemetic and gastroprokinetic drug metoclopramide is a weak substrate of the blood‐brain barrier (BBB) efflux transporter P‐gp and displays central nervous system (CNS) side effects (i.e., extrapyramidal symptoms and tardive dyskinesia) caused by dopamine D_2_ receptor blockade in the basal ganglia. These side effects occur with a higher incidence in elderly people. We used positron emission tomography to assess the brain distribution of [^11^C]metoclopramide in young (*n* = 11, 26 ± 3 years) and elderly (*n* = 7, 68 ± 9 years) healthy men both after administration of a microdose (9 ± 7 µg) and a microdose co‐injected with a therapeutic dose of unlabeled metoclopramide (10 mg). For both doses, elderly subjects had a significantly higher total volume of distribution (*V*_T_) of [^11^C]metoclopramide in the basal ganglia than young subjects (microdose: +26%, therapeutic dose: +41%). Increases in *V*_T_ (= *K*_1_/*k*_2_) were caused by significant decreases in the transfer rate constant of [^11^C]metoclopramide from brain into plasma (*k*_2_, microdose: −18%, therapeutic dose: −30%), whereas the distributional clearance from plasma into brain (*K*_1_) remained unaltered. This reduction in the clearance of [^11^C]metoclopramide (*k*_2_) from the brains of elderly subjects may be caused by an age‐related decrease in the activity of P‐gp at the BBB and may contribute to the higher incidence of CNS side effects of metoclopramide in the aged population. Our data suggest that an age‐associated decrease in the clearance properties of the BBB may modulate the CNS effects or side effects of clinically used P‐gp substrates.

**Study Highlights**

**WHAT IS THE CURRENT KNOWLEDGE ON THE TOPIC?**

☑ The antiemetic and gastroprokinetic drug metoclopramide is a weak substrate of the blood‐brain barrier (BBB) efflux transporter P‐gp and displays central nervous system (CNS) side effects (extrapyramidal symptoms, tardive dyskinesia), which occur with a higher incidence in elderly people.

**WHAT QUESTION DID THIS STUDY ADDRESS?**

☑ We performed [^11^C]metoclopramide positron emission tomography scans in young and elderly healthy men, both after administration of a microdose and therapeutic dose of metoclopramide, to investigate whether differences in brain distribution of metoclopramide may explain its CNS side effects.

**WHAT DOES THIS STUDY ADD TO OUR KNOWLEDGE?**

☑ Our data revealed for both investigated doses significantly higher brain distribution and decreased brain clearance of [^11^C]metoclopramide in the elderly, which may, at least partially, be caused by an age‐related reduction in the activity of P‐gp at the BBB.

**HOW MIGHT THIS CHANGE CLINICAL PHARMACOLOGY OR TRANSLATIONAL SCIENCE?**

☑ Our data suggest that an age‐associated decrease in the clearance properties of the BBB may at least partially contribute to variability in response to several clinically used, CNS‐targeted drugs, which are weak P‐gp substrates (e.g., certain antidepressants, antipsychotics, antiepileptic drugs, and opioids).

Metoclopramide is a frequently prescribed antiemetic and gastroprokinetic drug, which is included in the World Health Organization’s List of Essential Medicines.^1^ It exerts its antiemetic effect through inhibition of dopamine D_2_ receptors and serotonin 5‐HT_3_ receptors in the chemoreceptor trigger zone within the area postrema of the medulla oblongata.^2^ The area postrema is located within the brain on the floor of the forth ventricle, but is not protected by the blood‐brain barrier (BBB). Metoclopramide is a substrate of rodent and human P‐glycoprotein (P‐gp, encoded in humans by the *ABCB1* gene and in rodents by the *Abcb1a* and *Abcb1b* genes).^3^, ^4^ At the BBB, P‐gp is located in the luminal (blood‐facing) membrane of brain capillary endothelial cells, where it acts as a gate keeper to prevent the brain distribution of a plethora of drugs pertaining to different therapeutic classes.^5^ Metoclopramide is considered a weak P‐gp substrate, which shows appreciable brain distribution despite being transported by P‐gp at the BBB.^4^ This is supported by the occurrence of central nervous system (CNS) side effects associated with the use of metoclopramide (i.e., extrapyramidal movement disorders), which are mediated through antagonism at dopamine D_2_ receptors in the basal ganglia.^2^ The US Food and Drug Administration (FDA) and the European Medicines Agency (EMA) restricted the long‐term use of metoclopramide because of the risk for development of tardive dyskinesia, a potentially irreversible serious movement disorder.^2^ The incidence of tardive dyskinesia was found to increase with age.^6^, ^7^, ^8^

We have labeled metoclopramide with the positron‐emitting radionuclide carbon‐11 (^11^C)^9^ and used positron emission tomography (PET) to study the brain distribution of [^11^C]metoclopramide in healthy subjects without and with concurrent infusion of the P‐gp inhibitor cyclosporin A.^10^ These experiments showed that brain distribution of [^11^C]metoclopramide is modulated by P‐gp, as reflected by a 29% increase in the total volume of distribution (*V*_T_) in the brain following partial P‐gp inhibition. Based on these findings, it may be hypothesized that the CNS side effects of metoclopramide may be at least partly controlled by the activity of P‐gp at the BBB.^11^, ^12^ P‐gp activity at the BBB may be variable among individuals, for instance, due to drug‐drug interactions, genetic polymorphisms, or disease. Several lines of evidence have suggested that the abundance and activity of P‐gp at the human BBB decreases with increasing age, which could provide a mechanistic explanation for the higher incidence of central side effects of metoclopramide in the aged population.^13^, ^14^, ^15^, ^16^, ^17^

In the present study, we assessed the brain kinetics of [^11^C]metoclopramide with PET imaging in young and elderly healthy male subjects. The availability of an i.v. dosage form of metoclopramide for human use provided us with the unique possibility to not only study [^11^C]metoclopramide brain distribution at microdose levels (< 100 µg), as typically administered in PET experiments, but also with co‐injection of a therapeutic dose of unlabeled metoclopramide (10 mg).

## METHODS

This study was approved by the Ethics Committee of the Medical University of Vienna and regulatory authority (EudraCT 2017‐000989‐30). All subjects provided written informed consent before participating. Eleven young male subjects (mean age: 26 ± 3 years, mean weight: 75 ± 7 kg) and seven elderly male subjects (mean age: 68 ± 9 years, mean weight: 88 ± 15 kg) were included into the study. Subjects were judged as healthy based on clinical and neurological examination, routine blood and urine laboratory assessments, and urine drug screening. Medication known to not interact with the aims of the study was allowed (**Table** **S1**).

### Genotyping

Pre‐analytical procedures were performed by the MedUni Wien Biobank according to standard operating procedures in an International Organization for Standardization (ISO) 9001‐certified environment.^18^ The common *ABCB1* single‐nucleotide polymorphisms (SNPs) 2677G>T/A (rs2032582), 3435C>T (rs1045642), and 1236C>T (rs1128503) were assessed as described previously.^19^

### Radiotracer synthesis

[^11^C]Metoclopramide was synthesized as described before^20^ and formulated in sterile phosphate‐buffered saline solution containing 8.6% (v/v) ethanol.

### PET imaging and blood sampling

Subjects underwent two dynamic 60‐minute brain PET scans on an Advance (GE Healthcare, Waukesha, WI) stand‐alone PET scanner. For the first PET scan, a microdose (9 ± 7 µg) of [^11^C]metoclopramide was administered, and for the second PET scan [^11^C]metoclopramide was co‐injected with a therapeutic dose (10 mg) of unlabeled metoclopramide. For i.v. injection, [^11^C]metoclopramide was diluted to a final volume of 15 mL with physiological saline solution and administered as a constant bolus over 3 minutes with a Perfusor compact S (B. Braun AG, Melsungen, Germany). For the second scan, the contents of one Paspertin 10 mg ampoule (2 mL, Haupt Pharma Wuelfing GmbH, Germany) were added to the solution of [^11^C]metoclopramide in saline. The administered radioactivity amount was 334 ± 41 MBq for the first scan and 339 ± 51 MBq for the second scan. During each of the two scans, starting with [^11^C]metoclopramide injection, arterial blood samples (3 mL) were collected approximately every 10 seconds for the first 4 minutes followed by 9‐mL samples at 5, 10, 20, 30, 40, and 60 minutes after radiotracer injection. Aliquots of blood and plasma were measured for radioactivity in a gamma‐counter, which was cross‐calibrated with the PET camera. The plasma samples collected at 5, 10, 20, 30, and 40 minutes after radiotracer injection were analyzed for radiolabeled metabolites of [^11^C]metoclopramide with radio‐high performance liquid chromatography (radio‐HPLC), as described in detail elsewhere.^10^ Radio‐HPLC eluates were collected in 1‐minute fractions, which were counted for radioactivity in a gamma‐counter. The radio‐HPLC fractions from the 30 and 40‐minute time points in the second PET scan containing the radiolabeled metabolites of [^11^C]metoclopramide were pooled from different subjects and used to identify the chemical structure of the metabolites using liquid chromatography‐tandem mass spectrometry (LC‐MS/MS) as described in the **Supplementary Methods**. A mono‐exponential decay function was fitted to the percentage of parent [^11^C]metoclopramide in plasma vs. time and then applied to the corresponding decay‐corrected total radioactivity counts in plasma to derive a metabolite‐corrected arterial input function.

### Imaging data analysis

Individual T1‐weighted magnetic resonance images acquired on a MAGNETOM Skyra 3.0 Tesla scanner (Siemens Medical Solutions) were segmented with SPM12 (Statistical Parametric Mapping, Wellcome Trust Centre for Neuroimaging, UK) and the adult brain maximum probability map (“Hammersmith atlas”; n30r83) was used to define whole brain grey matter (WBGM) and a grey matter region comprising the left and right caudate nucleus, nucleus accumbens, putamen, thalamus, and pallidum (designated as basal ganglia (BG); mean volume: 19.7 ± 3.9 cm^3^) as regions of interest.^21^ Regions of interest were transferred to the respective dynamic PET data sets to extract concentration‐time curves. The PMOD Kinetic Modeling tool (PMOD version 3.6, PMOD Technologies, Zurich, Switzerland) was used to analyze the PET and metabolite‐corrected plasma data using a reversible 1‐tissue‐2‐rate constant compartmental model to estimate the rate constants for radioactivity transfer from plasma into brain (*K*_1_, mL/(cm^3^.min)) and from brain into plasma (*k*_2_, 1/min), and the total volume of distribution (*V*_T_ = *K*_1_/*k*_2_, mL/cm^3^).^10^ *V*_T_ equals the brain‐to‐plasma concentration ratio of [^11^C]metoclopramide at steady‐state. The fractional arterial blood volume in the brain (*V*_b_) was included as a fitting parameter.

For display purposes, the brain and plasma concentration‐time curves were expressed in units of percentage of the injected dose per cm^3^ or per mL (%ID/cm^3^ or %ID/mL). The area under the concentration‐time curves (AUC, %ID/cm^3^.min or %ID/mL.min) was calculated using Prism software (version 8.4.3; GraphPad Software, La Jolla, CA). To generate mean plasma concentration‐time curves, data were interpolated to the same time points in all subjects. For the therapeutic dose scan, %ID/mL values in plasma and %ID/cm^3^ values in the brain were converted into mass concentrations (µmol/L) by multiplication with the injected dose of unlabeled metoclopramide (in µmol) multiplied by 10.

### Statistical analysis

Our study was exploratory, so no sample size calculation was performed. Statistical analysis was performed using Prism software. After confirmation of the normal distribution of the data using the Shapiro–Wilk normality test, outcome parameters were compared for each scan between the young and elderly group using a two‐sided, unpaired *t*‐test, and within each group between scan 1 and scan 2 using a two‐sided, paired *t*‐test. The level of statistical significance was set to a *P* value of < 0.05. All values are given as mean ± SD.

## RESULTS

We included 11 young and 7 elderly subjects into our study. The study was prematurely terminated before inclusion of 10 elderly subjects due to dismantlement of the PET scanner. Subjects underwent two PET scans with [^11^C]metoclopramide: a first scan in which a microdose of metoclopramide was administered (9 ± 7 µg), and a second scan in which [^11^C]metoclopramide was co‐injected with a therapeutic dose of unlabeled metoclopramide (10 mg). One young subject (patient 15) underwent only the first, microdose PET scan. Adverse events occurring during the study are listed in **Table** **S1**. Two young and two elderly subjects experienced mild or moderate akathisia in response to therapeutic dose metoclopramide administration, which led in the case of the two elderly subjects to premature termination of the second PET scan within 15–20 minutes after radiotracer injection. Scan two data from these two elderly subjects (patient 26 and patient 29) were excluded from analysis. In addition, for one elderly subject (patient 22) the arterial cannula clotted at start of scan two, so that only venous blood sampling was possible. Scan two data from this subject were excluded from kinetic modeling analysis.

In all subjects, plasma samples collected at different time points after [^11^C]metoclopramide injection were analyzed for radiolabeled metabolites by radio‐HPLC. [^11^C]Metoclopramide appeared to be metabolized at a similar rate in the young and the elderly group, both for the microdose and therapeutic dose (**Figure** **S1**). At 40 minutes after [^11^C]metoclopramide injection, the fraction of total radioactivity in plasma corresponding to parent [^11^C]metoclopramide was, for the microdose, 0.49 ± 0.12 for the young and 0.48 ± 0.05 for the elderly group, and, for the therapeutic dose, 0.48 ± 0.10 for the young and 0.48 ± 0.10 for the elderly group. Two radiolabeled metabolites of [^11^C]metoclopramide were observed in plasma: the first (major) radiometabolite eluted on radio‐HPLC with a retention time of 3–4 minutes and the second (minor) radiometabolite with a retention time of 6–7 minutes (retention time parent [^11^C]metoclopramide: 8–9 minutes; **Figure** **S2**). Radio‐HPLC fractions from the therapeutic dose scan containing each radiolabeled metabolite were pooled and analyzed by LC‐MS/MS (**Figure** **S3**). Positive ion mass spectra of the first (major) radiometabolite showed a stable protonated (M + H) molecular ion of 492.22 amu with subsequent loss of 16 amu and 176 amu in agreement with the molecular weight of oxidative addition followed by conjugation with glucuronic acid (**Figure** **S3**). The concentration of the second (minor) radiometabolite was below the detection limit of the mass spectrometer and its structure could therefore not be identified.

**Figure 1:**
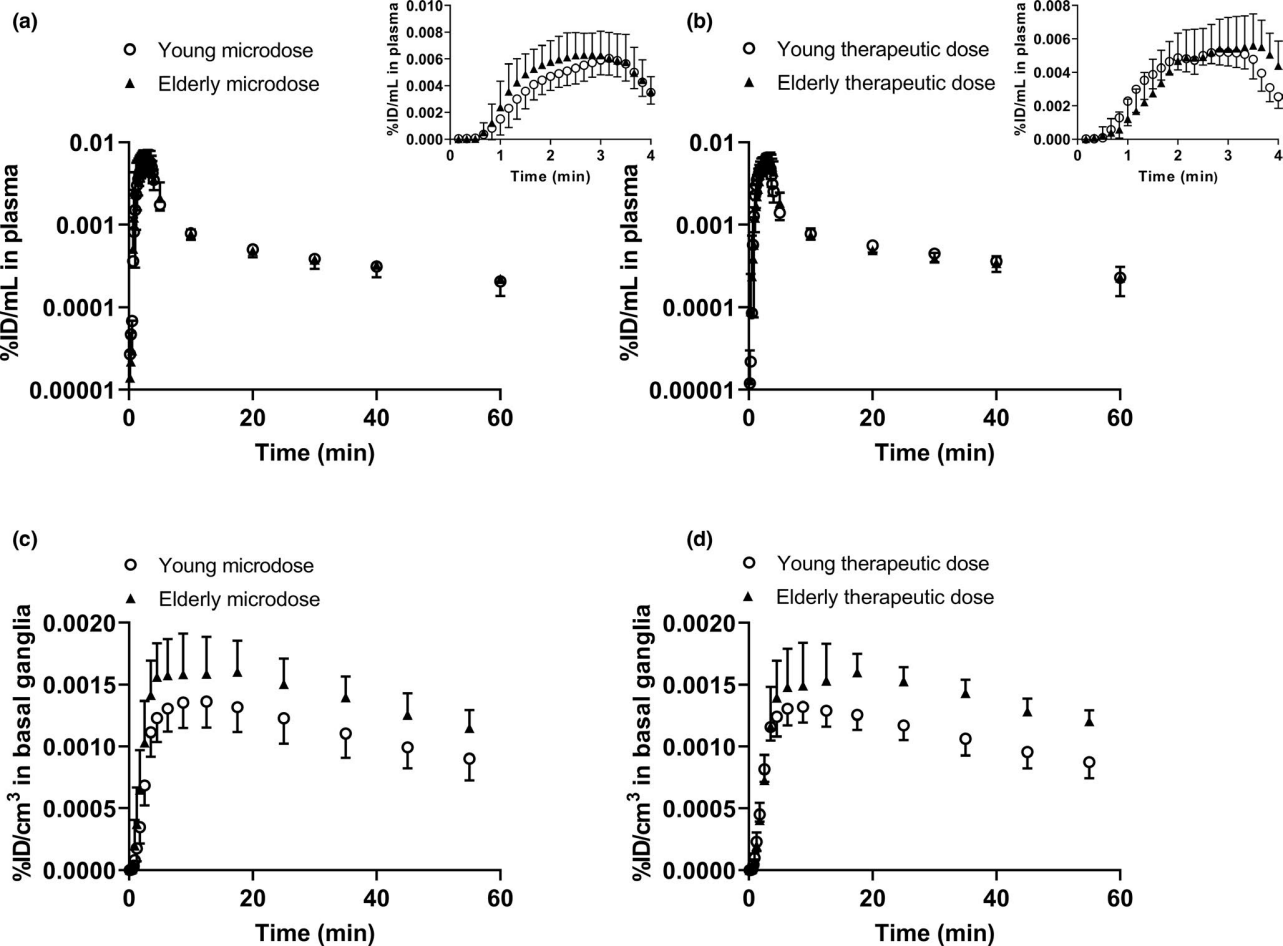
Concentration‐time curves (mean ± SD) of [^11^C]metoclopramide in arterial plasma (corrected for metabolites) (**a**, **b**) and in the basal ganglia (**c**, **d**) in young and elderly subjects for the microdose (**a**, **c**, young: *n* = 11, elderly: *n* = 7) and for the therapeutic dose (**b**, **d**, young: *n* = 10, elderly: *n* = 7). Inserts in **a** and **b** show the first 4 minutes of arterial plasma concentration‐time curves.

Arterial plasma concentration‐time curves of [^11^C]metoclopramide were comparable between young and elderly subjects for both administered doses (**Figure** **1a,b**). Peak arterial plasma concentration (C_max_) of [^11^C]metoclopramide and AUC_plasma_ calculated from 0 to 60 minutes after radiotracer injection were not significantly different between the young and the elderly group for both doses. The C_max_ was 0.0063 ± 0.0012 %ID/mL in young subjects and 0.0069 ± 0.0018 %ID/mL in elderly subjects for the microdose and 0.0060 ± 0.0009 %ID/mL (2.0 ± 0.3 µmol/L) in young subjects and 0.0061 ± 0.0008 %ID/mL (2.0 ± 0.3 µmol/L) in elderly subjects for the therapeutic dose. AUC_plasma_ was 0.042 ± 0.007 %ID/mL.min in young subjects and 0.045 ± 0.006 %ID/mL.min in elderly subjects for the microdose and 0.043 ± 0.007 %ID/mL.min in young subjects and 0.044 ± 0.006 %ID/mL.min in elderly subjects for the therapeutic dose. AUC_plasma_ values were not significantly different between the microdose and the therapeutic dose for the young and the elderly groups, respectively.

**Figure 2:**
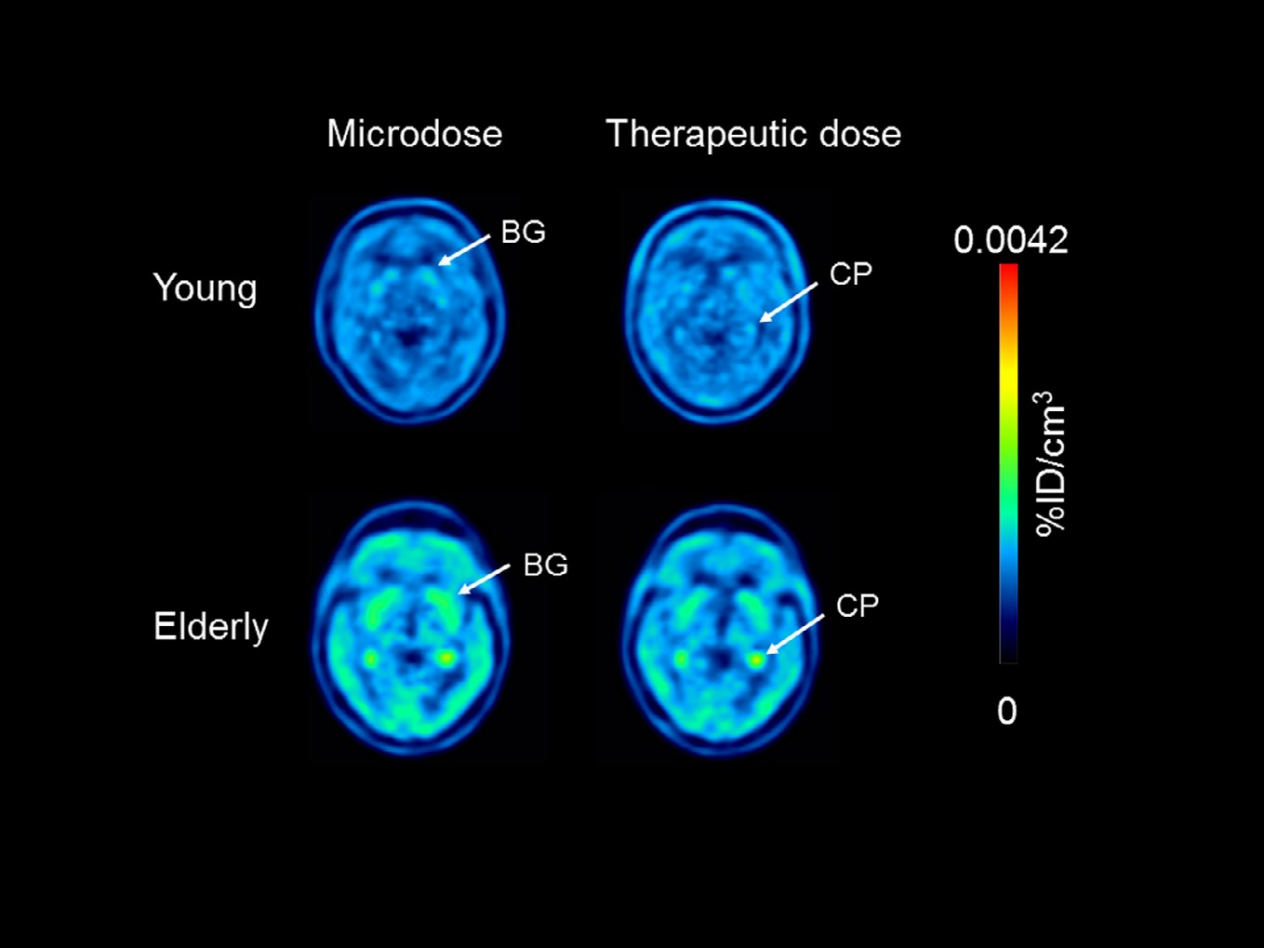
Representative positron emission tomography summation images (5–60 minutes) for one young (patient 16) and one elderly subject (patient 13) after injection of the microdose or therapeutic dose. Radioactivity concentration is expressed as percentage of the injected dose per cm^3^ (%ID/cm^3^). Anatomical structures are labeled with arrows, BG, basal ganglia; CP, choroid plexus.

For the brain, WBGM and the BG were analyzed as regions of interest. The latter were selected because they are rich in dopamine D_2_ receptors, which mediate the central adverse effects of metoclopramide. In **Figure** **1**, concentration‐time curves of [^11^C]metoclopramide in the BG are shown, and in **Figure** **2** representative PET summation images are shown. [^11^C]Metoclopramide concentrations in the BG were in the range of ~ 0.0015 %ID/cm^3^, which corresponded for the therapeutic dose scan to a mass concentration of ~ 0.5 µmol/L. In the BG, exposure to [^11^C]metoclopramide was significantly higher in the elderly than in the young group for both investigated doses (microdose: AUC, young: 0.061 ± 0.010 %ID/cm^3^.min, elderly: 0.076 ± 0.011 %ID/cm^3^.min, *p* = 0.014; therapeutic dose: AUC, young: 0.059 ± 0.006 %ID/cm^3^.min, and elderly: 0.077 ± 0.008 %ID/cm^3^.min, *p* = 0.0017).

**Figure 3:**
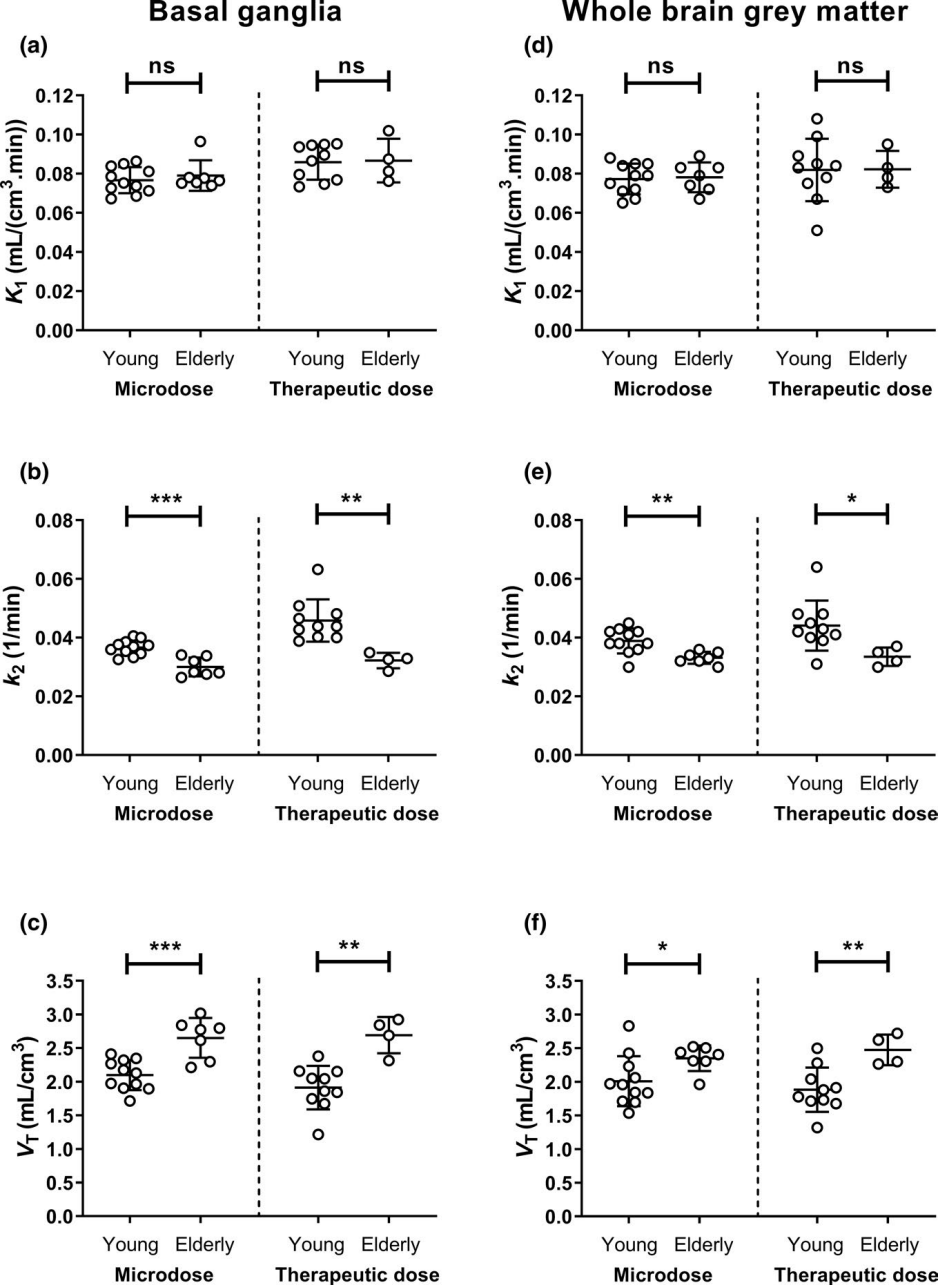
Outcome parameters from kinetic modeling (*K*_1_, **a**, **d**; *k*_2_, **b**, **e**; *V*_T_, **c**, **f**) for young and elderly subjects in the basal ganglia and whole brain grey matter after administration of the microdose or therapeutic dose. ns, not significant; *V*_T_, volume of distribution, *P < 0.05, **P < 0.01, ***P < 0.001, two‐sided, unpaired *t*‐test.

Brain PET data were analyzed by kinetic modeling using a 1‐tissue‐2‐rate constant model. The model outcome parameters *K*_1_, *k*_2_, and *V*_T_ are shown for individual subjects in **Figure** **3** both for BG and WBGM. Mean values of model outcome parameters from all studied groups are given in **Table** **1**. In both investigated brain regions, *V*_T_ was significantly higher (from + 17% to + 41%) for the elderly than for the young group, both for the microdose and the therapeutic dose (**Figure** **3c,f**). This increase in *V*_T_ (= *K*_1_/*k*_2_) in the elderly group was caused by a significant decrease in *k*_2_ (from −15% to −30%; **Figure** **3b,e**), whereas *K*_1_ remained unchanged (**Figure** **3a,d**). In WBGM, none of the model outcome parameters differed significantly between the microdose and the therapeutic dose, both for the young and the elderly group. In the BG of the young group, *K*_1_ and *k*_2_ were significantly higher for the therapeutic dose as compared with the microdose, but *V*_T_ was not different. *V*_b_ estimates were in similar range as the physiological value (0.05) and did not significantly differ between the young and the elderly group for both investigated doses (**Table** **1**).

**Table 1:** Model outcome parameters for the brain in young and elderly subjects after administration of a microdose or therapeutic dose of metoclopramide

Brain region	Group	*K*_1_ (mL/(cm^3^.min))	*k*_2_ (1/min)	*V*_T_ (mL/cm^3^)	*V*_b_
Basal ganglia	Young microdose	0.077 ± 0.007 (2 ± 0)	0.037 ± 0.003 (3 ± 1)	2.1 ± 0.2 (2 ± 1)	0.047 ± 0.009 (8 ± 3)
	Elderly microdose	0.079 ± 0.008 (2 ± 1)	0.030 ± 0.003 (4 ± 3)***	2.7 ± 0.3 (3 ± 2)***	0.045 ± 0.014 (7 ± 2)
	Young therapeutic dose	0.086 ± 0.009 (2 ± 0)	0.046 ± 0.007 (4 ± 0)	1.9 ± 0.3 (2 ± 0)	0.041 ± 0.013 (11 ± 5)
	Elderly therapeutic dose	0.087 ± 0.011 (3 ± 2)	0.032 ± 0.003 (5 ± 4)**	2.7 ± 0.3 (4 ± 2)**	0.057 ± 0.021 (7 ± 4)
Whole brain grey matter	Young microdose	0.077 ± 0.008 (2 ± 1)	0.039 ± 0.004 (4 ± 1)	2.0 ± 0.4 (2 ± 0)	0.059 ± 0.012 (8 ± 2)
	Elderly microdose	0.078 ± 0.008 (2 ± 1)	0.033 ± 0.002 (3 ± 1)**	2.3 ± 0.2 (2 ± 1)*	0.050 ± 0.017 (8 ± 7)
	Young therapeutic dose	0.082 ± 0.016 (2 ± 1)	0.044 ± 0.009 (4 ± 1)	1.9 ± 0.3 (2 ± 1)	0.063 ± 0.012 (9 ± 3)
	Elderly therapeutic dose	0.082 ± 0.009 (4 ± 3)	0.034 ± 0.003 (9 ± 6)*	2.5 ± 0.2 (6 ± 3)**	0.072 ± 0.027 (10 ± 5)

Values are reported as mean ± SD. The value in parentheses represents the precision of parameter estimates (expressed as their mean standard error in percent ± SD).

*K*_1_ (mL/(cm^3^.min)), distributional clearance of radioactivity from plasma into brain; *k*_2_ (1/min), rate constant for radioactivity transfer from brain into plasma; *V*_b_, fractional arterial blood volume in the brain; *V*_T_ (mL/cm^3^), total volume of distribution.

* *P* < 0.05, **P < 0.01, ***P < 0.001 for comparison of elderly group with respective young group using a two‐sided *t*‐test.

Subjects were genotyped for three common *ABCB1* SNPs (**Table** **S2**). One young (patient 20) and one elderly (patient 14) subject were homozygous carriers of the variant TTT haplotype, which has been linked to decreased P‐gp activity. Due to the small sample size, no statistical analysis could be performed, but model outcome parameters appeared to be similar in these two subjects as compared with the other subjects (**Table** **S2**).

## DISCUSSION

The aim of our study was to compare the brain kinetics of [^11^C]metoclopramide between young and elderly subjects in order to investigate whether the reported higher incidence of central side effects of metoclopramide in the aged population may be related to differences in the extent of its BBB transfer. In line with our hypothesis, we found that [^11^C]metoclopramide brain distribution was significantly higher in the elderly than in the young group. This included the BG, the brain region in which metoclopramide exerts its extrapyramidal side effects through dopamine D_2_ receptor antagonism.

We chose for our study a paradigm, in which the brain kinetics of [^11^C]metoclopramide were assessed in each study participant both after the administration of a microdose (9 ± 7 µg) and after the administration of a microdose co‐injected with a therapeutic dose of unlabeled metoclopramide (10 mg). The latter provided us with the unique possibility to monitor the brain concentrations of metoclopramide under conditions that are identical to its therapeutic usage.

We analyzed plasma samples collected from study participants during the PET scan by radio‐HPLC for the presence of radiolabeled metabolites of [^11^C]metoclopramide and used LC‐MS/MS to elucidate the structure of the major radiolabeled metabolite in plasma. This metabolite was identified as the corresponding *N*‐O glucuronide (**Figure** **S3**), which has also been detected in urine as a major metoclopramide metabolite after administration of a single oral dose of 20 mg to healthy volunteers.^22^ Metabolism of metoclopramide is mainly mediated by CYP2D6,^23^ which is a highly polymorphic enzyme. Consequently, variability in CYP2D6‐mediated metoclopramide metabolism may be linked to the occurrence of metoclopramide side effects.^24^ In our study, no differences in the rate of [^11^C]metoclopramide metabolism were observed between the young and the elderly group during the 1 hour period following i.v. drug administration (**Figure** **S1**). Moreover, the plasma pharmacokinetics of [^11^C]metoclopramide were comparable in the young and the elderly groups (**Figure** **1**). This suggested that age‐related differences in [^11^C]metoclopramide brain exposure were not caused by differences in peripheral [^11^C]metoclopramide disposition. Of note, the plasma pharmacokinetics of [^11^C]metoclopramide were similar for the microdose and the therapeutic dose over the short duration of the PET experiments, pointing to dose linearity of metoclopramide disposition within the studied 1,000‐fold dose range.

We found differences in [^11^C]metoclopramide brain exposure (AUC_brain_) between the young and the elderly groups. In the BG, AUC was 24% higher in the elderly group for the microdose and 29% higher for the therapeutic dose. Consistent with the higher brain exposure, two elderly subjects experienced moderate akathisia related to therapeutic dose metoclopramide administration, which led to premature termination of the second PET scan (**Table** **S1**).

Kinetic modeling provided mechanistic explanation for the increased brain exposure to [^11^C]metoclopramide in elderly subjects: the *V*_T_ parameter, which corresponds to the brain‐to‐plasma concentration ratio at steady‐state, was in the BG 26% higher in the elderly for the microdose and 41% higher for the therapeutic dose (**Figure** **3**, **Table** **1**). The increase in *V*_T_ (= *K*_1_/*k*_2_) in the elderly group was caused by a decrease in *k*_2_ and not by an increase in *K*_1_. This is in good agreement with results from our previous studies,^10^, ^25^ and suggests that the initial uptake of [^11^C]metoclopramide across the BBB (i.e., *K*_1_) was unaltered in the elderly group, whereas the clearance properties of the BBB were decreased (*k*_2_). It should be noted here that the used kinetic model cannot distinguish active transport from passive diffusion across the BBB. One plausible explanation for the decreased brain clearance of [^11^C]metoclopramide in the elderly is an age‐related reduction in the activity of P‐gp in brain capillary endothelial cells.^26^ As opposed to avid P‐gp substrates, which are transported to a high extent by P‐gp at the BBB and whose brain entrance is prevented by the action of P‐gp (influx hindrance), metoclopramide is a weak P‐gp substrate, which enters the brain despite being transported by P‐gp.^12^ The action of P‐gp lies in controlling the rate of elimination of metoclopramide from the brain (*k*_2_, efflux enhancement), which can be expected to have an effect on the occurrence of CNS side effects.^12^ Metoclopramide may resemble other CNS‐active drugs, which are only weak P‐gp substrates and whose therapeutic effects in the brain may be modulated by P‐gp (e.g., certain antidepressants, antipsychotics, antiepileptic drugs, and opioids).^12^ Similar to our findings in elderly subjects, a lower abundance of P‐gp at the BBB of infants, as revealed by semiquantitative immunohistochemical analysis,^27^, ^28^ may lead to higher metoclopramide brain exposure and contribute to the higher incidence of extrapyramidal side effects in this group, which has led to the restriction of metoclopramide usage in children who are younger than 1 year.^29^ It can, however, not be excluded that other factors than P‐gp contributed to the observed differences in [^11^C]metoclopramide brain distribution between the elderly and the young group (e.g., age‐related differences in protein/lipid binding of [^11^C]metoclopramide in the brain or differences in the passive permeability of the BBB).

Accumulation of [^11^C]metoclopramide appeared to be slightly higher in the BG than in the rest of the brain, which may point to binding to dopamine D_2_ receptors (**Table** **1**). Dopamine D_2_ receptor binding would be expected to be displaceable by co‐administration of unlabeled metoclopramide. The PET‐derived concentration of metoclopramide in the BG after i.v. injection of 10 mg was ~ 0.5 µmol/L, which is higher than the affinity constant of metoclopramide for dopamine D_2_ receptors (*K*_d_ = 46 nmol/L).^30^ However, there were no significant differences in *V*_T_ in the BG between the microdose and the therapeutic dose, both in the young and the elderly group, suggesting that binding of [^11^C]metoclopramide to dopamine D_2_ receptors was masked by nonspecific binding and contributed only to a negligible extent to the measured PET signal. An in‐depth assessment of the pharmacokinetic‐pharmacodynamic relationship of metoclopramide would therefore require a dedicated radiotracer to assess dopamine D_2_ receptor occupancy in the BG (e.g., [^11^C]raclopride).

The *ABCB1* gene is highly polymorphic with several different SNPs. Some of these SNPs have been associated with changes in P‐gp expression and response to CNS drugs, although there is considerable controversy about the functional significance of various *ABCB1* SNPs.^31^ The SNPs 2677G> T, 3435C> T and 1236C> T are the most common variants in the coding sequence. These SNPs are reported to be in strong linkage disequilibrium accounting for two major haplotypes, CGC and TTT. Variants 2677T, 3435T, and 1236T displayed decreased P‐gp activity *in vitro* in a substrate‐specific manner.^32^ We genotyped all study participants for these SNPs and identified one young and one elderly subject with the TTT haplotype. However, model outcome parameters did not markedly differ in these two subjects from the other subjects (**Table** **S2**).

[^11^C]Metoclopramide has been developed as a novel PET tracer to measure P‐gp activity at the BBB.^9^, ^10^, ^25^ As [^11^C]metoclopramide is only a weak P‐gp substrate, its baseline brain distribution is higher as compared with previously developed avid P‐gp substrate probes for PET, such as [^11^C]verapamil and [^11^C]*N*‐desmethyl‐loperamide.^12^ The brain distribution of (*R*)‐[^11^C]verapamil has been investigated in young and elderly subjects, which revealed moderately increased *V*_T_ values in WBGM (from + 15% to + 18%) in the elderly group.^13^, ^17^ Our present study revealed differences in [^11^C]metoclopramide *V*_T_ values in the examined brain regions between the young and the elderly (from + 17% to + 41% in the elderly), which were of similar magnitude as those for (*R*)‐[^11^C]verapamil.^13^, ^17^

In conclusion, our study provided evidence for a decreased clearance of metoclopramide from the brains of elderly subjects, which may be related to an age‐dependent reduction in cerebral P‐gp activity and which may contribute to the higher incidence of CNS adverse effects of metoclopramide in the elderly. An age‐associated decrease in the clearance properties of the BBB may exert an influence on the efficacy or safety of CNS‐targeted drugs. The results of our study may need further confirmation by performing PET studies with other weak P‐gp substrates.

## Funding

This work was supported by the Austrian Science Fund (FWF) (grant KLI 694‐B30, to O.L.).

## Conflict of Interest

The authors declared no competing interests for this work.

## Author Contributions

O.L. and M.B. wrote the manuscript. M.B., O.L., W.J., N.T., M.H., and M.Z. designed the research. M.B., V.P., K.B., M.W., S.B., A.M.‐S., A.T., and H.H. performed the research. M.B., O.L., K.B., W.J., and H.H. analyzed the data.

## Supporting information

Fig S1‐S3

Table S1

Table S2

Supplementary Material
