# Supplementary material for: Impaired Clearance From the Brain Increases the Brain Exposure to Metoclopramide in Elderly Subjects
Source: Clin Pharmacol Ther. 2020 Oct 14;109(3):754–61. doi: 10.1002/cpt.2052 (PMC7983943; doi:10.1002/cpt.2052)

**Supplementary Figure 1** Fraction of parent [^11^C]metoclopramide (mean ± SD) in arterial plasma over time in the young and elderly group for the microdose (**a**) and for the therapeutic dose (**b**).


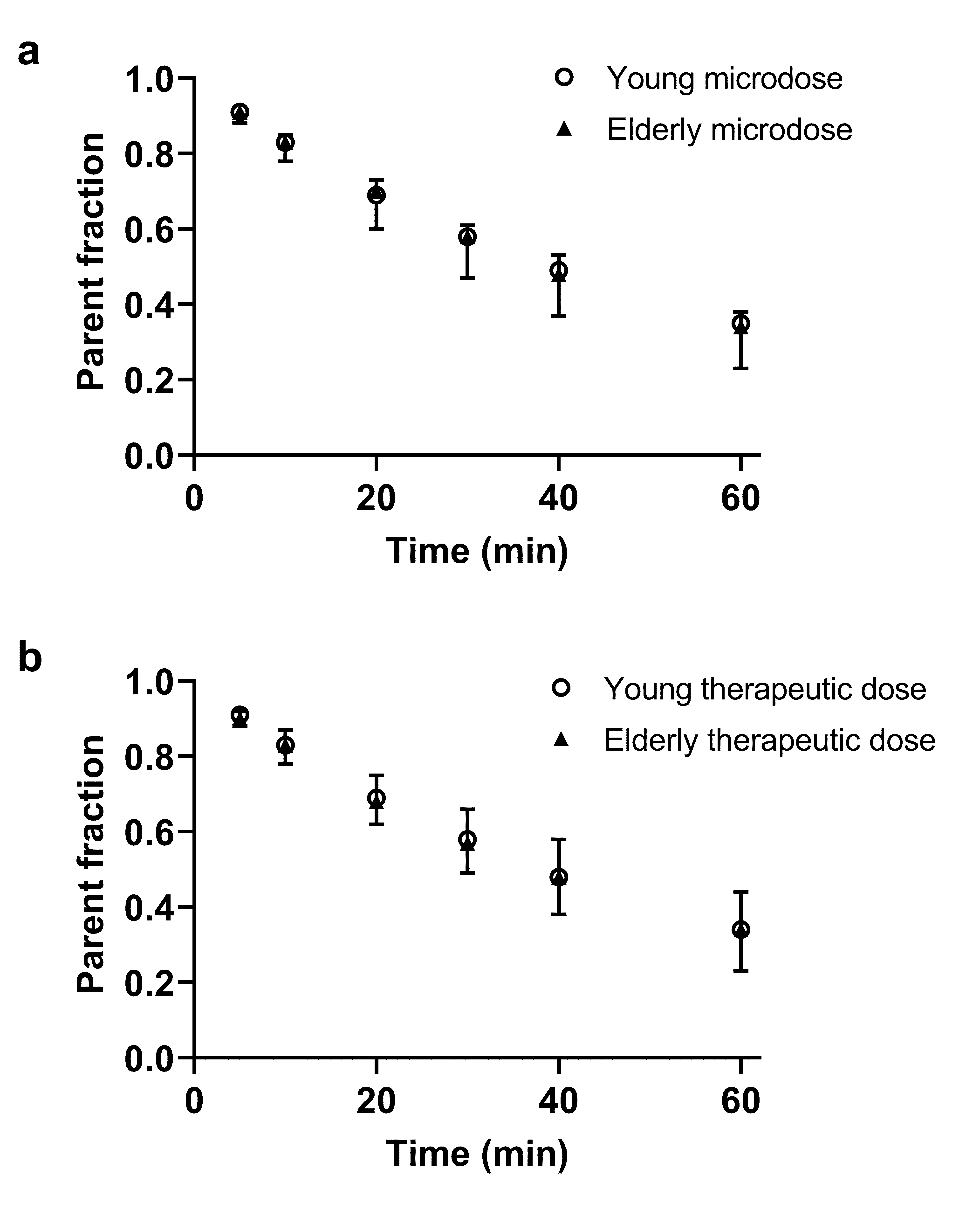


**Supplementary Figure 2** Radio-HPLC analysis of a plasma sample collected at 40 min after injection of [^11^C]metoclopramide co-injected with a therapeutic dose of unlabeled metoclopramide in one representative young subject (p24). Shown are decay-corrected counts in 1-min fractions collected from radio-HPLC eluate and measured offline in a gamma-counter. The first (main) radiometabolite eluted from 3-4 min and accounted for a fraction of 0.32 ± 0.07 of total radioactivity and the second (minor) radiometabolite eluted from 6-7 min and accounted for a fraction of 0.14 ± 0.04 of total radioactivity (mean of all subjects in both scans for the 40 min time point).


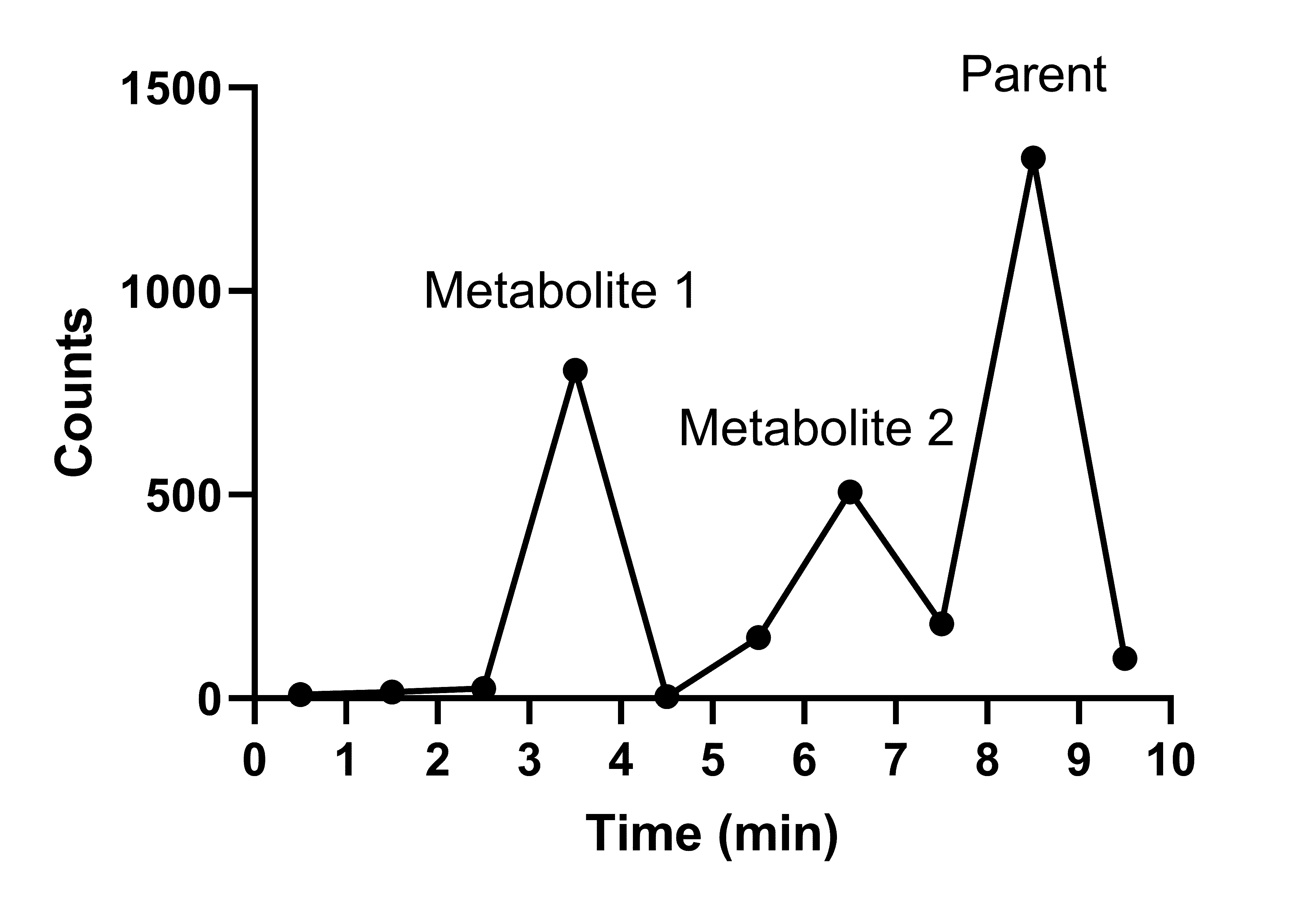


**Supplementary Figure 3** MS/MS spectrum for the main radiolabeled metabolite of [^11^C]metoclopramide. Ions indicated by * denote unchanged fragmentation compared with parent metoclopramide using static CID energy at 30 ev. A fragmentation at m/z 300 indicated the main metabolite before oxidation and glucuronidation, and at m/z 492 oxidation and glucuronidation. Fragments ions at m/z 273, 227, 184 and 143 serve as a confirmation of the identity of this metoclopramide metabolite (m/z 300).


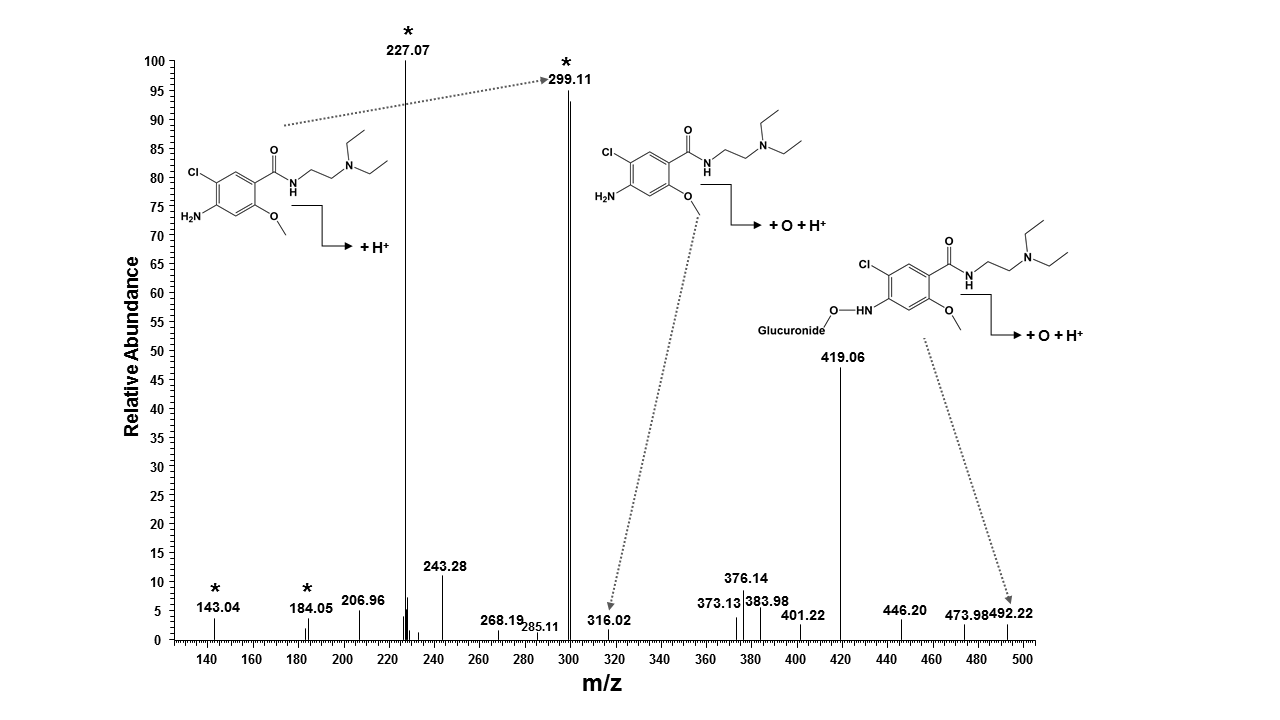

Supplement: Supplementary file 1 — Fig S1‐S3 [file CPT-109-754-s004.docx]
