# Supplementary material for: Impaired Clearance From the Brain Increases the Brain Exposure to Metoclopramide in Elderly Subjects
Source: Clin Pharmacol Ther. 2020 Oct 14;109(3):754–61. doi: 10.1002/cpt.2052 (PMC7983943; doi:10.1002/cpt.2052)
Supplement: Supplementary file 2 — Table S1 [file CPT-109-754-s003.docx]

**Supplementary Table 1** **Adverse events (AE) recorded during the study and concomitant medication used during the study**

| **Subject code** | **Adverse events** | **Relation to meto-clopramide (10 mg)** | **Severity classification** | **Medication** |
| --- | --- | --- | --- | --- |
| **Young** | | | | |
| p12 | - | - | - | - |
| p15 | - | - | - | - |
| p16 | - | - | - | - |
| p17 | Increased peristaltic movements | Possibly related | Mild | - |
| p18 | Akathisia | Related | Mild | - |
| p19 | Allergic reaction (heat sensation on whole body, difficulties with breathing) | Possibly related | Moderate | - |
| p20 | Panic attack | Possibly related | Mild | - |
| p21 | - | - | - | Pregabalin 75 mg od |
| p23 | Akathisia | Related | Mild | - |
| p24 | - | - | - | - |
| p25 | Restlessness | Possibly related | Mild | - |
| **Elderly** | | | | |
| p13 | - | - | - | Olmesartan+amlodipine+hydrochloro-thiazide 40+10+25 mg od; atenolol 25 mg bd; urapidil 60 mg on demand |
| p14 | Hematoma arterial cannulation site (left wrist) | Unrelated | Mild | Tamsulosin retard 0.4 mg od |
| p22 | Hypertension during PET scan | Probably related | Mild | - |
| p26 | Akathisia *^a^* | Related | Moderate | Escitalopram 10 mg every second day od; flavonoids 500 mg bd |
| p27 | - | - | - | - |
| p28 | - | - | - | - |
| p29 | Akathisia *^a^* | Related | Moderate | - |

od, once daily, bd, twice daily

*^a^* Second PET scan had to be terminated at 15-20 min after start of [^11^C]metoclopramide/metoclopramide injection due to AE
