# Supplementary material for: Impaired Clearance From the Brain Increases the Brain Exposure to Metoclopramide in Elderly Subjects
Source: Clin Pharmacol Ther. 2020 Oct 14;109(3):754–61. doi: 10.1002/cpt.2052 (PMC7983943; doi:10.1002/cpt.2052)
Supplement: Supplementary file 3 — Table S2 [file CPT-109-754-s002.docx]

**Supplementary Table 2** **Subjects’ age, *ABCB1* genotype and whole brain grey matter *V*_T_ and *k*_2_ values for the microdose (scan 1) and for the therapeutic dose (scan 2)**

| Subject code | Age  (years) | *ABCB1* Genotype | | | *V*_T_ scan 1  (mL/cm^3^) | *V*_T_ scan 2  (mL/cm^3^) | *k*_2_ scan 1  (1/min) | *k*_2_ scan 2  (1/min) |
| --- | --- | --- | --- | --- | --- | --- | --- | --- |
|  |  | **2677G>T/A**  **(rs2032582)** | **3435C>T**  **(rs1045642)** | **1236C>T**  **(rs1128503)** |  |  |  |  |
| p12 | 25 | [T];[G] | [C];[T] | [C];[T] | 1.8 | 2.0 | \| 0.043 \| \| --- \| | \| 0.043 \| \| --- \| |
| p15 | 24 | [T];[A] | [C];[T] | [C];[T] | 1.7 | - | \| 0.041 \| \| --- \| | - |
| p16 | 23 | [G];[T] | [C];[T] | [C];[T] | 2.0 | 1.7 | \| 0.045 \| \| --- \| | \| 0.048 \| \| --- \| |
| p17 | 23 | [G];[G] | [C];[T] | [C];[C] | 2.1 | 1.9 | \| 0.038 \| \| --- \| | \| 0.045 \| \| --- \| |
| p18 | 28 | [G];[G] | [C];[T] | [C];[C] | 2.0 | 1.7 | \| 0.038 \| \| --- \| | \| 0.031 \| \| --- \| |
| p19 | 27 | [G];[T] | [C];[T] | [C];[T] | 2.2 | 2.5 | \| 0.038 \| \| --- \| | \| 0.040 \| \| --- \| |
| p20 | 28 | [T];[T] | [T];[T] | [T];[T] | 1.8 | 1.8 | \| 0.036 \| \| --- \| | \| 0.042 \| \| --- \| |
| p21 | 28 | [G];[T] | [C];[T] | [C];[T] | 2.8 | 1.3 | \| 0.030 \| \| --- \| | \| 0.064 \| \| --- \| |
| p23 | 31 | [G];[A] | [C];[C] | [C];[C] | 1.5 | 1.9 | \| 0.042 \| \| --- \| | \| 0.041 \| \| --- \| |
| p24 | 26 | [T];[T] | [T];[T] | [C];[T] | 1.7 | 1.7 | \| 0.042 \| \| --- \| | \| 0.039 \| \| --- \| |
| p25 | 27 | [G];[T] | [C];[C] | [C];[T] | 2.4 | 2.3 | \| 0.035 \| \| --- \| | \| 0.048 \| \| --- \| |
| p13 | 76 | [T];[G] | [C];[T] | [C];[T] | 2.4 | 2.6 | \| 0.030 \| \| --- \| | \| 0.030 \| \| --- \| |
| p14 | 60 | [T];[T] | [T];[T] | [T];[T] | 2.5 | 2.3 | \| 0.035 \| \| --- \| | \| 0.037 \| \| --- \| |
| p22 | 65 | [G];[T] | [C];[T] | [C];[T] | 2.4 | - | \| 0.032 \| \| --- \| | - |
| p26 | 80 | [G];[G] | [C];[T] | [C];[C] | 2.3 | - | \| 0.036 \| \| --- \| | - |
| p27 | 76 | [G];[T] | [C];[T] | [C];[T] | 2.5 | 2.3 | \| 0.033 \| \| --- \| | \| 0.032 \| \| --- \| |
| p28 | 61 | n.d. | n.d. | n.d. | 2.3 | 2.7 | \| 0.032 \| \| --- \| | \| 0.035 \| \| --- \| |
| p29 | 60 | [G];[T] | [C];[T] | [C];[T] | 2.0 | - | \| 0.034 \| \| --- \| | - |

*V*_T_ (mL/cm^3^), total volume of distribution in whole brain grey matter; *k*_2_ (1/min), rate constant for radioactivity transfer from whole brain grey matter into plasma;

n.d. not determined
