# Supplementary material for: Impaired Clearance From the Brain Increases the Brain Exposure to Metoclopramide in Elderly Subjects
Source: Clin Pharmacol Ther. 2020 Oct 14;109(3):754–61. doi: 10.1002/cpt.2052 (PMC7983943; doi:10.1002/cpt.2052)
Supplement: Supplementary file 4 — Supplementary Material [file CPT-109-754-s001.docx]

**Metabolite identification using LC-MS/MS**

Metabolite identification in human plasma was performed using a Dionex Ultimate 3000 ultrahigh-performance liquid chromatograph (UHPLC) connected with a LTQ-XL linear ion trap mass spectrometer (Thermo Fisher Scientific, Inc. Waltham, MA, USA). Radio-HPLC fractions obtained from the analysis of plasma samples in the therapeutic dose [^11^C]metoclopramide PET scan and containing the two observed radiolabeled metabolites of [^11^C]metoclopramide were pooled and concentrated to dryness. Dry residues were dissolved in 0.5 mL of water/acetonitrile (1:1) and injected onto the UHPLC-column (injected volume: 90 µL). Separation of metoclopramide metabolites was carried out at 40°C using an Acclaim 120 C18 column (120Å, 5µm, 150 x 2.1 mm; Thermo Scientific Scientific, Inc.) at a flow rate of 0.5 mL/min. The mobile phase consisted of a continuous gradient mixed from 0.1% aqueous formic acid (mobile phase A) and 0.1% formic acid in acetonitrile (mobile phase B). The gradient ranged from 5% B at 0 min where it remained constant for 2 min. Subsequently, the percentage of B was increased to 50% at 14 min, increased further to 90% at 15 min, and was kept constant at 95% until 19 min. The percentage of B was then decreased within 2 min to 5% to equilibrate the column for 5 min before application of the next sample. The mass spectrometer was operated in the positive ionization mode using a HESI source (300°C heater temperature, 40/10/1 arb. units for the sheath, aux and sweep N_2_ gas, respectively, and 3.2 kV spray voltage at 275°C transfer-capillary temperature). MS scans were performed with an m/z range from 300 to 900 and a resolution of 5000 units (at m/z = 300). MS/MS scans of the 3 most abundant ions were achieved through collision-induced dissociation (CID) fragmentation at 30% static normalized collision energy.
